# Será que a Medição da Big Endotelina-1 Pode Ter um Papel em Pacientes Admitidos Por Enfarte Agudo do Miocárdio sem Supra Desnivelamento do Segmento ST?

**DOI:** 10.36660/abc.20230013

**Published:** 2023-03-01

**Authors:** João Presume

**Affiliations:** 1 Serviço de Cardiologia Hospital de Santa Cruz Centro Hospitalar Lisboa Ocidental Lisboa Portugal Serviço de Cardiologia, Hospital de Santa Cruz, Centro Hospitalar Lisboa Ocidental, Lisboa – Portugal; 2 Comprehensive Health Research Centre NOVA Medical School Universidade Nova de Lisboa Lisboa Portugal Comprehensive Health Research Centre, NOVA Medical School, Universidade Nova de Lisboa, Lisboa – Portugal

**Keywords:** Infarto do Miocárdio sem Supradesnivelamento do Segmento ST, Doença da Arterial Coronariana, Endotelina-1, Big-Endotlina-1/tendências

A Endotelina-1 (ET-1) é o principal membro da família dos peptídeos da endotelina e é uma das substâncias vasoconstritoras mais potentes conhecidas até o momento.^1^ Atua através dos receptores ETA, promovendo vasoconstrição, inflamação e proliferação celular.^1,2^ Vários tipos celulares sintetizam essa molécula, principalmente as células endoteliais vasculares e musculares lisas, macrófagos e fibroblastos, resultantes da clivagem da big endotelina-1 (big ET-1).^3^ Esta última é uma molécula intermediária do sistema da endotelina, que tem uma meia-vida mais longa (apesar de menor atividade biológica), sendo um marcador mais adequado para a quantificação da atividade do peptídeo endotelina.^4^

Neste estudo transversal, os investigadores avaliaram os níveis de big ET-1 em uma coorte de pacientes com infarto do miocárdio sem elevação do segmento ST (IAMSSST).^5^ No geral, 766 pacientes foram incluídos, e o objetivo principal foi explorar a relação entre esse marcador e a carga de doença coronariana avaliada pelo escore SYNTAX.^6^ Depois de estratificar a coorte em 3 grupos de acordo com esse escore (baixo, intermediário e alto), eles descobriram que os níveis medianos de ET-1 eram progressivamente mais altos conforme a doença coronariana tornou-se mais complexa (0,30 pmol/L, 0,41 pmol/L e 0,58 pmol/L, respectivamente). Eles também descobriram que esse marcador tinha uma correlação ncom o escore SYNTAX (r=0,378), que alcançou significância estatística, e encontrou boa discriminação na identificação de pacientes com escores moderados a altos (AUC 0,695).^5^

Estes resultados alinham-se com a fisiopatologia do sistema endotelínico, uma vez que estas moléculas parecem ter um papel particular no desenvolvimento da disfunção endotelial, tendo sido implicadas em várias doenças cardiovasculares, como hipertensão arterial e aterosclerose.^1,7,8^ Além disso, quando se trata de doença arterial coronária, foi demonstrado que a presença de IM está associada a níveis mais altos de ET-1 e que níveis mais altos desse marcador foram associados a maior mortalidade a longo prazo em pacientes com infarto do miocárdio com supradesnivelamento do segmento ST (IAMCSST).^8,9^

Os peptídeos de endotelina são biomarcadores não invasivos e fáceis de obter que podem ser particularmente interessantes para pacientes com infarto do miocárdio.^10^ Em primeiro lugar, a possibilidade de predizer um escore SYNTAX mais alto pode ajudar na estratificação de pacientes com IAMSSST. Níveis elevados desses biomarcadores podem apontar para uma abordagem invasiva mais precoce, uma vez que está associada a doença arterial coronariana previsivelmente mais complexa, com maior risco isquêmico. Em segundo lugar, a maior probabilidade de a doença coronária ser mais indicada para revascularização cirúrgica pode ter implicações na escolha da terapêutica antitrombótica, especialmente a dupla antiagregação plaquetária, o que pode levar a um atraso da referida intervenção.

Apesar desses resultados e implicações relevantes, este estudo teve algumas limitações. Além de ser um estudo retrospectivo de centro único, que incluiu apenas pacientes chineses, a correlação entre grandes níveis de ET-1 e o escore SYNTAX foi razoável. Além disso, e como esperado, os subgrupos não foram homogêneos após a estratificação de acordo com esse escore. A população com maior carga de doença coronariana também apresentou mais marcadores de risco (mais velhos, classe Killip mais alta, fração de ejeção mais baixa e escore GRACE mais alto), que também são aspectos importantes na previsão da complexidade da doença. No entanto, o ponto de corte mais bem identificado de grandes níveis de ET-1 (0,35 pmol/L) ainda foi um preditor independente de escore SYNTAX moderado/alto, mesmo após o controle desses fatores significativos (OR 2,962).

Relativamente às perspectivas futuras, estes resultados deverão suscitar novas investigações sobre o valor deste marcador como indicador de estratificação precoce do risco invasivo, nomeadamente em estudos prospectivos. Além disso, esse biomarcador e sua relação com o escore SYNTAX também podem ter um papel importante em pacientes com síndromes coronarianas crônicas, pois podem auxiliar na seleção de pacientes para angiografia invasiva precoce devido a um possível risco aumentado de doença aterosclerótica mais grave.

Em suma, a big ET-1 pode ser mais uma peça que pode ajudar a prever a carga de doença aterosclerótica coronariana e orientar o manejo desses pacientes, como evidenciado por este interessante estudo.
