# Supplementary material for: Será que a Medição da Big Endotelina-1 Pode Ter um Papel em Pacientes Admitidos Por Enfarte Agudo do Miocárdio sem Supra Desnivelamento do Segmento ST?
Source: Arq Bras Cardiol. 2023 Mar 1;120(2):e20230013. [Article in Portuguese] doi: 10.36660/abc.20230013 (PMC12080702; doi:10.36660/abc.20230013)
Supplement: Translation

# Translation: Can the Measurement of Big Endothelin-1 Have a Role in Patients Admitted Due to Non-ST Segment Elevation Myocardial Infarction?

**Authors:** João Presume

**Affiliations:** 1 Portugal Serviço de Cardiologia, Hospital de Santa Cruz, Centro Hospitalar Lisboa Ocidental, Lisboa – Portugal; 2 Portugal Comprehensive Health Research Centre, NOVA Medical School, Universidade Nova de Lisboa, Lisboa – Portugal

**Keywords:** Non-ST Elevated Myocardial Infarction, Coronary Artery Disease, Endothelin-1, Big-Endothelin-1/trends

Endothelin-1 (ET-1) is the main member of the endothelin peptide family and is one of the most potent vasoconstrictor substances known to date.^1^ It acts through ET_A _receptors, promoting vasoconstriction, inflammation, and cell proliferation.^1,2^ Several cell types synthesize this molecule, particularly the vascular endothelial and smooth muscle cells, macrophages, and fibroblasts, resulting from the cleavage of big endothelin-1 (big ET-1).^3^The latter is an intermediate molecule from the endothelin system, which has a longer half-life (despite lower biological activity), being a more appropriate marker for the quantification of endothelin peptide activity.^4^

In this cross-sectional study, investigators evaluated the levels of big ET-1 in a cohort of patients with non-ST segment elevation myocardial infarction (NSTEMI).^5^ Overall, 766 patients were included, and the main objective was to explore the relationship between this marker and coronary disease burden as assessed by the SYNTAX score.^6^ After stratifying the cohort into 3 groups according to this score (low, intermediate, and high), they found that the median big ET-1 levels were progressively higher as the coronary disease became more complex (0.30 pmol/L, 0.41 pmol/L, and 0.58 pmol/L, respectively). They also found that this marker had a fair correlation with the SYNTAX score (r=0.378), which met statistical significance, and found good discrimination in identifying patients with moderate-high scores (AUC 0.695).^5^

These results align with the pathophysiology of the endothelin system since these molecules seem to have a particular role in developing endothelial dysfunction, having been implicated in various cardiovascular diseases such as arterial hypertension and atherosclerosis.^1,7,8^ Furthermore, when it comes to coronary artery disease, it has been demonstrated that the presence of MI is associated with higher levels of ET-1 and that higher levels of this marker were associated with higher long-term mortality in patients with ST-elevation myocardial infarction (STEMI).^8,9^

Endothelin peptides are non-invasive, easy-to-obtain biomarkers that can be particularly interesting to patients with myocardial infarction.^10^ Firstly, the possibility of predicting a higher SYNTAX score may help in the stratification of patients with NSTEMI. High levels of these biomarkers may point towards an earlier invasive approach since it is associated with predictably more complex coronary artery disease, with higher ischemic risk. Secondly, the higher probability of coronary disease being more suitable for surgical revascularization may have implications regarding antithrombotic therapy choice, especially double antiplatelet therapy, which may lead to a delay of said intervention.

Despite these relevant results and implications, this study had some limitations. Besides being a retrospective, single-center study, which only included Chinese patients, the correlation between big ET-1 levels and SYNTAX score was only fair. Moreover, and as expected, subgroups were not homogeneous after stratification according to this score. The population with higher coronary disease burden also had more risk markers (older, higher Killip class, lower ejection fraction, and higher GRACE score), which are also important aspects of predicting disease complexity. Nevertheless, the best-identified cut-off of big ET-1 levels (0.35 pmol/L) was still an independent predictor of moderate/high SYNTAX score, even after controlling for these significant factors (OR 2.962).

Regarding future perspectives, these results should elicit further investigations regarding the value of this marker as an indicator for earlier invasive risk stratification, namely in prospective studies. Moreover, this biomarker and its relationship with the SYNTAX score may also have an important role in patients with chronic coronary syndromes since it may aid in selecting patients for earlier invasive angiography due to a possible increased risk for more severe atherosclerotic disease.

All in all, big ET-1 might be yet another piece that may help predict coronary atherosclerotic disease burden and guide the management of these patients, as evidenced by this interesting study.
